# Erratum to: Complete genome sequence of *Atopobium parvulum* type strain (IPP 1246^T^)

**DOI:** 10.4056/sigs.992408

**Published:** 2010-06-15

**Authors:** Alex Copeland, Johannes Sikorski, Alla Lapidus, Matt Nolan, Tijana Glavina, Del Rio, Susan Lucas, Feng Chen, Hope Tice, Sam Pitluck, Jan-Fang Cheng, Rüdiger Pukall, Olga Chertkov, Thomas Brettin, Cliff Han, Cheryl Kuske, David Bruce, Lynne Goodwin, Natalia Ivanova, Konstantinos Mavromatis, Natalia Mikhailova, Amy Chen, Krishna Palaniappan, Patrick Chain, Manfred Rohde, Markus Göker, James Bristow, Jonathan A. Eisen, Victor Markowitz, Philip Hugenholtz, Nikos C. Kyrpides, Hans-Peter Klenk, John C. Detter

**Affiliations:** 1 DOE Joint Genome Institute, Walnut Creek, California, USA; 2 DSMZ - German Collection of Microorganisms and Cell Cultures GmbH, Braunschweig, Germany; 3 Los Alamos National Laboratory, Bioscience Division, Los Alamos, New Mexico, USA; 4 Biological Data Management and Technology Center, Lawrence Berkeley National Laboratory, Berkeley, California, USA; 5 Lawrence Livermore National Laboratory, Livermore, California, USA; 6 HZI - Helmholtz Centre for Infection Research, Braunschweig, Germany; 7 University of California Davis Genome Center, Davis, California, USA

## Correction to figure 1

In Copeland *et al*. [1], Figure 1 should appear as shown below. The mature cell shape has previously been described as cocci that occur singly, in pairs, in clumps, and in short chains, occasionally with central swelling [2,3]. Our paper showed cocci or coccobacilli, which is not the correct morphology for this species and was caused by a mix-up with the Figure 2 Pukall et al, [4].

**Figure 1:**
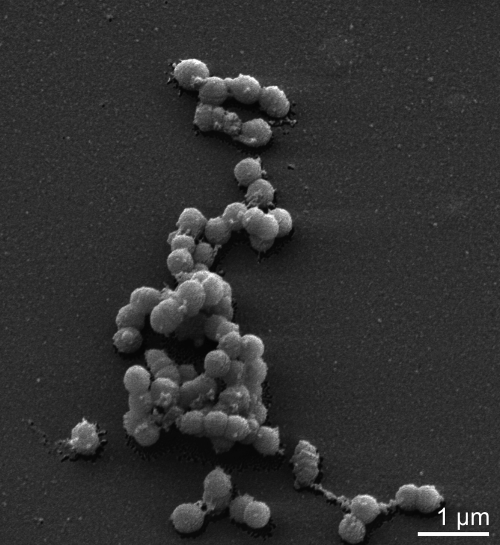
Scanning electron micrograph of *A. parvulum* IPP 1246^T^
